# Antidiabetic Activities of Hydromethanolic Leaf Extract of* Calpurnia aurea *(Ait.) Benth. Subspecies* aurea *(Fabaceae) in Mice

**DOI:** 10.1155/2018/3509073

**Published:** 2018-09-09

**Authors:** Yaschilal Muche Belayneh, Eshetie Melese Birru

**Affiliations:** ^1^Department of Pharmacy, College of Medicine and Health Sciences, Wollo University, P.O. Box 1145, Dessie, Ethiopia; ^2^Department of Pharmacology, School of Pharmacy, College of Medicine and Health Sciences, University of Gondar, P.O. Box 196, Gondar, Ethiopia

## Abstract

Diabetes mellitus is one of the largest global health problems demanding preventive and new therapeutic interventions. Currently, there is a need for safe, effective, and less costly antidiabetic medications, and investigating medicinal plants for new antidiabetic medication is an interesting research area. Thus, the present study was done to evaluate the antidiabetic activities of 80% methanolic leaf extract of* Calpurnia aurea *(Ait.) Benth. subspecies* aurea* (Fabaceae) in mice. Hypoglycemic and antihyperglycemic activity of the three doses (100mg/kg, 200 mg/kg, and 400 mg/kg) of crude hydromethanolic leaf extract was studied on normoglycemic, oral glucose loaded, and streptozotocin-induced diabetic mice models. The effect of the extract on body weight and diabetic dyslipidemia was also studied on streptozotocin-induced diabetic mice. Glibenclamide (5 mg/kg) was used as a standard drug in all cases. A glucose meter and an automated chemistry analyzer were used to measure blood glucose and serum lipid level respectively. Data were analyzed using one-way analysis of variance followed by Tukey's post hoc multiple comparison test. All the three doses of the plant extract (100mg/kg, 200 mg/kg, and 400 mg/kg) showed a significant (p<0.05) antihyperglycemic activity in the diabetic mice at the 7th and 14th day of repeated daily dose administration as compared to the negative diabetic control. But, the extract did not show significant blood glucose lowering activity in normoglycemic, oral glucose loaded, and diabetic mice after single dose administration, and it did not significantly improve the body weight loss and diabetic dyslipidemia of diabetic mice after repeated daily dose administration for 14 days. This study revealed that the hydromethanolic extract of* Calpurnia aurea* leaves possesses significant antihyperglycemic activity justifying the traditional use of the plant for diabetes.

## 1. Background

Diabetes mellitus (DM) is a metabolic disorder characterized by chronic hyperglycemia with abnormal carbohydrate, fat, and protein metabolism due to defects in insulin secretion, insulin action, or both [1]. The estimated number of people aged between 20 and 79 years with diabetes worldwide in 2015 was 415 million [2]. The global prevalence of DM is expected to rise to 552 million by 2030 [3]. Nearly 5 million people aged between 20 and 79 years died from diabetes in 2015, one death every six seconds. It was estimated that 12% of the global health expenditure was spent on diabetes in 2015 [2]. Globally, at least USD 376 billion was spent on DM in 2010, and this global expenditure is expected to reach USD 490 billion in 2030 [4]. Diabetes mellitus can directly affect serum lipid levels causing diabetic dyslipidemia which is one of its complications [5]. Diabetic dyslipidemia is mainly characterized by higher serum levels of triglyceride (TG), lower high density lipoprotein cholesterol (HDL-C), and high small dense LDL levels [6, 7].

Developing new antidiabetic medications from plant derived compounds which are easily accessible seems highly attractive research area as currently available medications have limitations in terms of safety, efficacy, and cost [8]. Globally, there are more than 1000 plant species that are being used as folk medicine for DM [8]. One of the plant families with the most potent hypoglycemic activity is Fabaceae, also called Leguminoseae [9]. In Ethiopia,* Calpurnia aurea* (Ait.) Benth. subspecies* aurea* (Fabaceae) is traditionally used for the treatment of DM. Peoples of shenasha, Agew, and Amhara in northwest Ethiopia use the leaf as well as the seed of the plant orally for the treatment of DM [10]. Similarly, people of Nekemtae town (east Wollega, Ethiopia) use leaf decoction of the plant orally to treat DM [11]. However, the antidiabetic activity of this medicinal plant is not scientifically validated.

Studies have shown that plant derived isoflavones possess hypoglycemic activity [12–14]. Isoflavones have been isolated from* Calpurnia aurea* (Ait.) Benth. subspecies* aurea* [15] indicating the plant may have blood glucose lowering activity. Additionally, the antidiabetic activity of medicinal plants is mainly due to the presence of alkaloids, phenolic compounds, flavonoids, and terpenoids [8, 9, 16–18]. The hydromethanolic extract of* Calpurnia aurea* leaves also contains these secondary metabolites known to have blood glucose lowering activity according to previous preliminary phytochemical studies [19, 20].

Induction of oxidative stress is a key process in the pathogenesis of DM and its complications [21–23], and the role of antioxidants in treating diabetes and its complications through prevention of oxidative stress has been explained in different studies [22–24]. Interestingly, previous studies revealed* Calpurnia aurea* leaves have strong* in vitro* antioxidant activities [25, 26] indicating the plant may possess antidiabetic activity. Thus, the present study was done to evaluate the antidiabetic activity of 80% methanolic leaf extract of* Calpurnia aurea* (Ait.) Benth. subspecies* aurea* (Fabaceae) in mice.

## 2. Materials and Methods

### 2.1. Drugs, Chemicals, and Instruments

Methanol absolute (Nice Chemicals, India), streptozotocin (Sigma Aldrich, Germany), glibenclamide (Julphar Pharmaceuticals, Ethiopia), citric acid monohydrate (Lab Tech Chemicals, India), tri-sodium citrate dihydrate (Blulux Labratories, India), sterilized water for injections (Nirma Ltd., India), 40% glucose solution (Reyoung Pharmaceuticals, China), analytical balance, pH meter, i-QARE DS-W® blood glucose meter, and strips (Alliance International, Taiwan), distilled water, mindray BS-240 clinical chemistry analyzer (Shenzhen Mindray Bio-Medical Electronics Co., Ltd,, China) were all of analytical grade.

### 2.2. Plant Material Collection and Preparation

Fresh leaves of* Calpurnia aurea* were collected from south Gondar zone of Amhara region, northwest Ethiopia in August 2017. After collection, taxonomic identification and authentication were done and the specimen of the plant was kept at the Herbarium of Biology Department, University of Gondar, with a voucher number YM001 for future reference.

### 2.3. Preparation of Plant Material Extract

The leaves of the plant were first thoroughly washed with distilled water and allowed to dry under shade with optimal ventilation. The dried leaves were then chopped to coarse powder. Nine hundred gram of the coarse powdered plant material was macerated in 80% methanol for 72 hours and then the extract was filtered using Whatman filter paper No. 1. Then, the residue was remacerated two times with fresh solvent, each for 72 hours, and the filtrates obtained from the successive maceration were dried in a hot air oven at 40 degree centigrade. The dried extract was then kept in a desiccator to maintain dryness till used in the experiment.

### 2.4. Experimental Animals

Healthy Swiss albino mice (weighing 25-30 g and age of 8-12 weeks) were purchased from the Ethiopian public health institute, Addis Ababa. The animals were then kept in the animal house of Department of Pharmacology, University of Gondar, using polypropylene cages. The animals were maintained under standard conditions (12 h light and 12 h dark cycle) and allowed free access to standard pellet laboratory diet and water* ad libitum*. Animals were acclimatized to the laboratory conditions for 1 week before the initiation of the experiment.

### 2.5. Acute Toxicity Study

Acute oral toxicity test was done based on the limit test recommendations of OECD No 425 Guideline [27]. On the first day of the test, one female Swiss albino mouse fasted for 3 hours was given 2000 mg/kg of the crude extract orally. Then the mouse was kept under strict observation for physical or behavioral changes for 24 h, with special attention during the first 4 hours. Because mortality was not observed in the first mouse, other four female mice fasted for 3-4 hours were sequentially given a single dose of 2000 mg/kg of the leaf extract and then observed in the same manner. The observation was continued for a total of 14 days for any sign of toxicity and mortality.

### 2.6. Grouping and Dosing of Animals

Male animals were used in all animal models (normoglycemic mice, oral glucose loaded mice, single dose treated diabetic mice, and repeated dose treated diabetic mice) because females are also less sensitive to insulin [28], and they are less sensitive to STZ compared to male animals [29, 30].

In the normoglycemic, oral glucose loaded, and single dose treated diabetic animal models, mice were randomly divided into five groups (6 mice per group). In all cases, Group I (negative control) was treated with 10 ml/kg distilled water (DW); Groups II, III, and IV were treated with 100 mg/kg, 200 mg/kg, and 400 mg/kg plant extract, respectively, whereas Group V (positive control) was treated with the standard drug, glibenclamide (5 mg/kg).

In the repeated daily dose treated diabetic animal model, mice were randomly divided into six groups (5 groups of diabetic mice and 1 additional group of normal mice, 6 mice per group). Group I (diabetic control) was treated with 10 ml/kg DW; Group II, III, and IV (diabetic test groups) were treated with 100 mg/kg, 200 mg/kg, and 400 mg/kg plant extract, respectively; Group V (diabetic positive control group) was treated with 5 mg/kg glibenclamide, whereas Group VI (normal control) was treated with 10 ml/kg DW.

Glibenclamide (5 mg/kg) was selected as a standard drug based on reports of previous studies [31–33]. The three doses of the plant extract were determined based on the result of the acute oral toxicity study. Oral route of administration was used in the study because people traditionally use the plant material orally [10, 11]. All the doses were given using an oral gavage after dissolving the plant extract in distilled water at a volume not exceeding 10 ml/kg body weight of the mouse [27].

### 2.7. Measurement of Blood Glucose Level

Blood samples were withdrawn from the tail vein of each animal aseptically, and blood glucose level (BGL) was measured using i-QARE DS-W® blood glucose meter. In all cases, BGL measurement was done in triplicate and the average value was taken.

### 2.8. Induction of Experimental Diabetes

Streptozotocin (STZ) was used to induce experimental diabetes. STZ was first dissolved in 0.1 M cold citrate buffer (pH=4.5). Then, the freshly prepared solution was given intraperitonially to the mice at a dose of 150 mg/kg [34]. Mice were fasted overnight for 16 hours prior to STZ administration. Food and water were allowed to the animals thirty minutes after the administration of STZ. Six hours after STZ administration, animals were allowed to drink 5% glucose solution for the next 24 hours to prevent hypoglycemic shock and death. Four days after STZ injection, animals were screened for diabetes. Mice with fasting blood glucose level > 200 mg/dl were included in the study as diabetic [32, 35]. Immediately after screening, STZ-induced diabetic animals were assigned randomly into different groups to perform the experiment.

### 2.9. Assessing Hypoglycemic Activity of the Extract in Normoglycemic Mice

Overnight (for 16 hours) fasted mice were randomly divided into five different groups (each group containing six animals). Then, the animals were treated according to their respective grouping as mentioned above. BGL of each mouse was measured just before treatment (at 0 hr) as baseline, and then at 1, 2, 4, and 6 hours after treatment.

### 2.10. Evaluating Effect of the Extract on Oral Glucose Tolerance in Normal Mice

Overnight fasting increases insulin dependent glucose utilization specifically in mice [36, 37]. Thus, it is logical to use mice for the oral glucose tolerance test in order to have a sensitive animal model for screening antihyperglycemic activity of the plant extract. After overnight fasting for 16 hours, mice were randomly divided into 5 groups (each group containing six animals). Then, animals were treated with distilled water, plant extract, and glibenclamide according to their respective grouping as mentioned above. Thirty minutes following each administration [31, 38], 2.5 g/kg glucose in a form of solution (40% w/v) was given orally to each animal [31]. BGL was measured for each animal just before treatment (at 0 minutes) as baseline, and then at 30, 60, and 120 minutes following the oral glucose load [31, 39].

### 2.11. Evaluating Antihyperglycemic Activity of Single Dose of the Extract in Streptozotocin-Induced Diabetic Mice

Overnight fasted (for 16 hours) diabetic mice were assigned randomly into 5 groups (each group containing 6 animals). Then, mice were treated with distilled water, plant extract, and glibenclamide according to their respective grouping. BGL was measured just before treatment (at 0 hr) as baseline, and then at 2, 4, 6, and 8 hours after treatment.

### 2.12. Assessing Antihyperglycemic Activity, Effect on Body Weight, and Antidyslipidemic Activity of Repeated Daily Doses of the Extract in Streptozotocin-Induced Diabetic Mice

After overnight fasting for 16 hours, STZ-induced diabetic mice and normal mice were randomly assigned into 6 groups (5 groups of diabetic mice and 1 group of normal mice, 6 animals per group). Then, mice were treated with distilled water, plant extract, and glibenclamide once daily for 14 days according to their respective grouping as explained above. Blood glucose level and body weight of mice were measured just before starting treatment on the 1st day of treatment (four days after STZ injection) as baseline and then on the 7th and 14th day of treatment following overnight fasting for 16 hours [40]. On day 15, overnight fasted mice were first sacrificed using overdose of an anesthetic, sodium pentobarbitone at a dose of 150 mg/kg IP, and then blood samples were collected from each animal in a sterile gel tube via cardiac puncture [33, 40]. The blood samples were left at room temperature for 2 hours and then centrifuged. The supernatant was immediately separated from the pellet to prepare serum samples in order to determine the level of triglyceride (TG), total cholesterol (TC), and high density lipoprotein cholesterol (HDL-C) using automated chemistry analyzer.

### 2.13. Ethical Considerations

The proposal of the study was submitted and approved by the Ethical Review Committee of the School of Pharmacy, University of Gondar, before the commencement of the study (ethical approval number, SOP4/77/09). The experiment was conducted in accordance with the Guide for the Care and Use of Laboratory Animals [41].

### 2.14. Statistical Analysis

All the data were expressed as mean ± standard error of the mean (SEM). Between and within group analysis were carried out using one-way ANOVA followed by Tukey's post hoc multiple comparison test. The results were considered to be significant when the P-value was less than 0.05. SPSS Version 20 software was used for data processing and analysis.

## 3. Result

### 3.1. Percentage Yield of Plant Material Extraction

A total of 154 grams of dried dark-brown gummy extract was harvested at the end of the extraction process. The extract was found to be better soluble in water than organic solvent. The percentage yield of the extract was found to be 17.11% (w/w).

### 3.2. Acute Oral Toxicity Study

The acute toxicity study of* Calpurnia aurea *leaf extract (CALE) did not show mortality in the animals at the limit dose of 2000 mg/kg during the observation period. Thus, the median lethal dose (LD50) of the leaf extract is greater than 2000 mg/kg. Besides, the toxicity study of CALE did not reveal any signs of toxicity: behavioral, neurological, autonomic, or physical changes.

### 3.3. Hypoglycemic Activity of the Hydromethanolic Leaf Extract in Normoglycemic Mice

The effect of hydromethanolic extract on fasting blood glucose level of normal mice is summarized in a table (Table 1). Between groups analysis revealed no significant difference in baseline fasting BGL across groups. All the three groups treated with different doses of CALE did not show a statistically significant reduction in BGL at all time points compared to the negative control group. But, it was found that BGL was significantly reduced by glibenclamide (5 mg/kg) at the 2nd (p<0.05), 4th (p<0.01), and 6th (p<0.05) hours compared to the negative control. Similarly, comparing GLC treated group with extract treated groups, it was revealed that 5 mg/kg GLC significantly reduced the BGL at the 2nd and 4th hours (p<0.05) compared to 100 mg/kg CALE treated group; at the 2nd hr (p<0.05) compared to 200 mg/kg CALE treated group; at the 2nd (p<0.01), 4th (p<0.05), and 6th (p<0.05) hours compared to the 400 mg/kg CALE treated group. There was no statistically significant difference in BGL when groups treated with different doses of the leaf extract were compared with each other at all time points.

Within group analysis showed that treatment with all the three doses of the extract and distilled water did not significantly reduce the BGL at all time points compared to the respective baseline level. But, the standard dug (glibenclamide) reduced the BGL significantly at the 2nd (p<0.05), 4th (p<0.001), and 6th (p<0.001) hours compared to the baseline level with percentage reduction, 32.37%, 40.63%, and 46.61%, respectively.

### 3.4. Antihyperglycemic Activity of the Hydromethanolic Leaf Extract of* Calpurnia aurea *in Oral Glucose Loaded Mice

There was no significant difference in baseline BGL across groups just before the administration of DW, CALE and glibenclamide (Table 2). Between groups analysis showed that all doses of CALE did not show a significant reduction in hyperglycemia at all time points compared to the negative control, whereas 5 mg/kg GLC reduced the hyperglycemia significantly at the 1st (p<0.05) and 2nd (p<0.001) hours after glucose administration compared to the vehicle treated group. Comparing the GLC treated group with plant extract treated groups, 5 mg/kg GLC significantly reduced the hyperglycemia at the 2nd hour (p<0.05) compared to 200 mg/kg CALE. There was no statistically significant difference in BGL at all time points when all the three plant extract treated groups were compared with each other.

Within a group analysis revealed that oral glucose loading caused a statistically significant (p<0.001) increment in BGL after 30 minutes in all groups compared to the baseline fasting BGL regardless of the treatments given. Additionally, significant hyperglycemia was observed at 1 hr after glucose load in all groups, except GLC (5 mg/kg) treated group, compared to the respective baseline BGL. But there was no statistically significant difference in BGL at the 2nd hour compared to the baseline level in all groups. Besides, significant reduction in BGL was observed at 60 and 120 minutes in all groups including the negative control compared to the respective BGL at 30 minutes after glucose administration.

### 3.5. Antihyperglycemic Activity of Single Dose of the Hydromethanolic Leaf Extract of* Calpurnia aurea *in Streptozotocin-Induced Diabetic Mice

Between and within group analysis were performed to see BGL differences across the various groups and time points, respectively (Table 3). The between group analysis indicated no significant difference in baseline fasting BGL across all groups. Similarly, there was no significant difference in BGL across all groups at the 2nd hour after treatment. Compared to the negative control, plant extract treated groups did not show a statistically significant reduction in BGL at all time points. Similarly, there was no significant difference in BGL at all time points when groups treated with plant extract were compared to each other and compared to the positive control.

Within a group comparison showed that there was no significant BGL reduction observed in CALE 100 mg/kg, CALE 200 mg/kg, and CALE 400 mg/kg treated groups at all time points compared to the baseline fasting BGL. However, percent reduction in BGL was recorded as 32.58% in CALE 100 mg/kg treated group, 21.59% in CALE 200 mg/kg treated group, and 22.75% in CALE 400 mg/kg treated group at the 8th hour compared to the respective baseline fasting level. The standard drug (glibenclamide, 5 mg/kg) produced a significant BGL reduction at the 4th, 6th, and 8th (p<0.001) hours compared to the initial level.

### 3.6. Antihyperglycemic Activity of the Repeated Daily Doses of the Hydromethanolic* Calpurnia aurea *Leaf Extract in Streptozotocin-Induced Diabetic Mice

**Figure 1:**
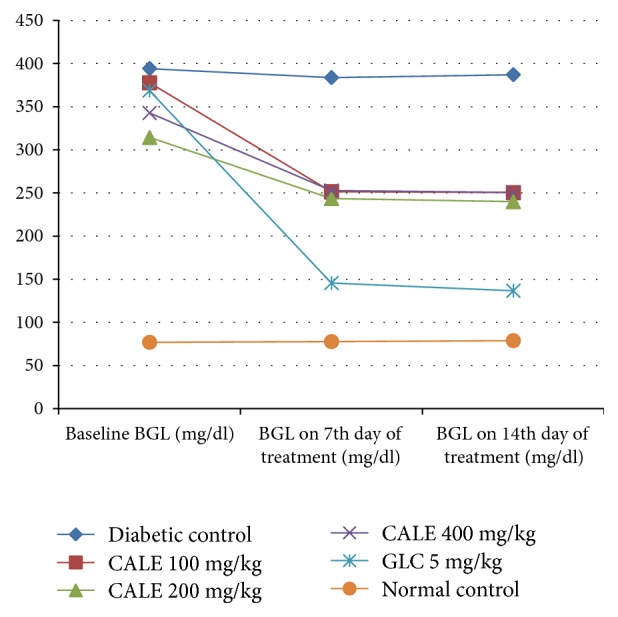
Effect of repeated daily doses of* Calpurnia aurea* leaf extract on blood glucose level of diabetic mice. CALE =* Calpurnia aurea* leaf extract, GLC = glibenclamide, and BGL = blood glucose level.

The effect of repeated daily doses of the plant extract on blood glucose level of diabetic mice is shown in Figure 1. Between group analysis indicated no significant difference in baseline fasting BGL across all groups of diabetic mice, but the baseline BGL of the diabetic groups was significantly (p<0.001) higher than the baseline BGL of the normal control (Table 4). All the three doses of CALE significantly (P<0.05) reduced the BGL on the 14th day of treatment compared to the diabetic control. The GLC treated group also showed significant reduction in blood glucose level on the 7th and 14th day of treatment compared to the diabetic control. But, GLC treated group showed no significant difference in BGL at all time points when compared to plant extract treated groups. There was no statistically significant difference in BGL at all time points when groups treated with plant extract were compared with each other.

Within a group analysis revealed that 100 mg/kg CALE significantly (P<0.05) reduced the BGL on the 14th day of treatment compared to the baseline level, but the diabetic control and the normal control did not show a significant change in BGL on 7th and 14th days compared to the respective baseline level. The standard drug reduced the BGL significantly (P<0.001) on the 7th and 14th days compared to the baseline level.

### 3.7. Effect of the Repeated Daily Doses of the Hydromethanolic Leaf Extract of* Calpurnia aurea *on Body Weight of Diabetic Mice

There was no significant difference in body weight of mice across all groups including the normal control just before induction of DM with STZ (Table 5). STZ produced significant loss of body weight in the diabetic control on the 7th and 14th day of treatment compared to the normal control. It was revealed that all the three doses of CALE (100, 200, and 400 mg/kg) showed no significant improvement in body weight at the 7th and 14th day of treatment compared to the diabetic control, but glibenclamide significantly improves the body weight loss of STZ-induced diabetic mice on the 14th day of treatment as compared to the diabetic control.

Intragroup analysis was done to compare the baseline body weight which was measured just before starting treatment with body weight at the 7th and 14th days of treatment. It was found that groups treated with the three doses of CALE (p<0.05) and the diabetic control (p<0.01) showed significant body weight reduction at the 14th day of treatment compared to the respective baseline body weight.

### 3.8. Effect of the Repeated Daily Doses of the Extract on Serum Lipid Level of Streptozotocin-Induced Diabetic Mice

There was a significant (p<0.001) elevation of serum total cholesterol and triglycerides, whereas there was significant reduction (p<0.001) of HDL cholesterol in the diabetic control compared to the normal control (Table 6). Administration of all the three doses of CALE for 14 days slightly reduced the level of serum total cholesterol and triglyceride while slightly increasing the HDL cholesterol level compared to the diabetic control, but the effect was not found to be statistically significant. The standard drug (glibenclamide) significantly reduced (p<0.001) the serum cholesterol and triglyceride level while increasing (p<0.01) the HDL cholesterol. There was no significant difference in the level of serum TC, TG, and HDL-C when groups treated with plant extract were compared with each other.

## 4. Discussion

Diabetes is one of the largest global health emergencies of the 21^st^ century [2]. There is a need for safer, more effective, and less costly treatment as currently available drug regimens of DM have limitations. Novel compounds with pan-target antidiabetic activity and long-term safety should be targeted for patients with coexisting diabetes and dyslipidemia. Thus, investigating plant derived compounds for DM is an attractive research area as they are believed to be safe and easily accessible and do not require laborious pharmaceutical synthesis [8, 40].

Previous acute oral toxicity tests were done on the hydromethanolic extract of* Calpurnia aurea* leaves, although there is discrepancy among the results of the studies [19, 20, 42]. The present study revealed that the median lethal dose (LD50) of the plant extract is greater than 2000 mg/kg showing a wide margin of safety.

Streptozotocin [2-deoxy-2-(3-methyl-3-nitrosourea)-1-D-glucopyranose] induced diabetes in mice is a known and well-documented model of experimental diabetes [43]. STZ is a better diabetogenic agent than alloxan with greater reproducibility and wider species effectiveness due to its better stability in aqueous solution before and after injection in animals [44]. DNA methylation, nitric oxide, and reactive oxygen species production are the major mechanisms associated with pancreatic *β* cell death secondary to STZ exposure [44]. Studies showed that single intraperitoneal injection of STZ at a dose of 150 mg/kg can produce sustained hyperglycemia in mice at least for a period of 8 weeks [45]. Similarly, 150 mg/kg STZ induced persistent hyperglycemia in our study with no significant change in BGL during the study period of two weeks as observed in the diabetic control.

In the present study, there were no significant differences in baseline BGL across groups as observed in all animal models. Similarly, the vehicle treated groups did not show detectable reduction in BGL compared to the baseline level in all animal models. But, significant BGL reduction was observed in the diabetic mice after repeated daily dose administration of the hydroalcoholic leaf extract, indicating the change in BGL was attributed to the treatment received.

The antidiabetic activity of medicinal plants is due to the presence of phenolic compounds, alkaloids, terpenoids, and flavonoids [8, 9, 16–18]. Thus, the antihyperglycemic activity of CALE may be due to the presence of these different secondary metabolites known to have blood glucose lowering activity with possible synergistic effects.

Antihyperglycemic activity was observed in glibenclamide treated diabetic mice, and glibenclamide produces its effect via selective blockage of ATP sensitive K^+^ channels (K_ATP_) in the plasma membrane of *β*-cells of the pancreas; thereby it leads to cytosolic depolarization and release of endogenous insulin [46]. This suggests that single dose intraperitoneal administration of STZ at a dose of 150 mg/Kg did not cause complete destruction of *β*-cells.

The antihyperglycemic activity of CALE may be due to potentiation of insulin effect either by increasing the secretion of insulin from beta cells of pancreas or by increasing the peripheral glucose uptake [47]. However, detailed molecular studies are required to identify the exact mechanism for the antihyperglycemic activity of CALE observed in the study.

Induction of experimental diabetes using STZ causes severe body weight loss in mice [29, 33]. Studies have shown that severe hyperglycemia in mice is associated with an increased body weight loss after STZ treatment [29, 45]. Similarly, our study revealed that STZ-induced diabetes caused significant body weight loss in the diabetic control. The induction of diabetes with STZ leads to loss of body weight due to increased wasting of fat stores [48], muscle, and tissue proteins [49, 50]. Lipid abnormality is also one of the complications of diabetes mellitus, manifested mainly by high serum TG, TC, and low HDL-C [7, 33]. Insulin deficiency causes activation of hormone sensitive lipase that can lead to increased lipolysis and increased secretion of VLDL from the liver [6, 7]. Decreased activity of lipoprotein lipase, secondary to insulin deficiency, also leads to decreased clearance of chylomicrons and VLDL [51]. In addition, hypertriglyceridemia stimulates the enzymatic action of cholesteryl ester transfer protein which leads to an increase in triglyceride content of LDL and HDL. Triglyceride-enriched HDL particles easily undergo catabolism, and Triglyceride-enriched LDL particles undergo subsequent hydrolysis via hepatic lipase or lipoprotein lipase resulting in reduced LDL particle size [7].

## 5. Conclusion

This study revealed that the hydromethanolic extract of* Calpurnia aurea* leaves has significant antihyperglycemic activity, but it did not significantly improve body weight loss and diabetic dyslipidemia in diabetic animals.

## Figures and Tables

**Table 1:** Hypoglycemic activity of hydromethanolic leaf extract of* Calpurnia aurea* in normoglycemic mice.

**Group**	**Blood glucose level (mg/dl)**				
	**0 hr**	**1 hr**	**2 hr**	**4 hr**	**6 hr**
DW 10ml/kg	68.17 ± 7.97	70.22 ± 7.90	72.33 ± 8.09	73.5 ± 7.33	68.06 ± 7.99
CALE 100mg/kg	70.67 ± 4.36	73.78 ± 2.76	72.61 ± 3.42	66.33 ± 2.08	64.00 ± 3.88
CALE 200mg/kg	68.83 ± 4.14	73.83 ± 4.08	71.56 ± 4.59	65.56 ± 2.56	56.33 ± 4.92
CALE 400mg/kg	76.72 ± 3.21	79.11 ± 5.96	83.17 ± 3.89	67.17 ± 5.29	67.39 ± 6.61
GLC 5mg/kg	70.56 ± 5.86	54.00 ± 5.34	47.72 ± 5.03^a1 b1 c1 d2 *β*1^	41.89 ± 2.73^a2 b1 d1 *β*3^	37.67 ± 2.4^a1 d1 *β*3^

Each value represents mean ± SEM; n=6 for each treatment. ^**a**^Compared to the negative control, ^**b**^compared to CALE 100mg/kg, ^**c**^compared to CALE 200mg/kg, ^**d**^compared to CALE 400mg/kg, and ^**β**^compared to baseline blood glucose level. ^1^p < 0.05, ^2^p < 0.01, and ^3^p < 0.001. CALE = *Calpurnia aurea* leaf extract, DW = distilled water, and GLC = glibenclamide.

**Table 2:** Effect of *Calpurnia aurea* leaf extract on oral glucose tolerance in normal mice.

**Group **	**Blood glucose level (mg/dl)**			
	**0 min**	**30 min **	**60 min**	**120 min**
DW 10 ml/kg	87.06 ± 8.25	202.95 ± 15.84^*β*3^	142.17 ± 15.66^*β*1 *µ*1^	112.45 ± 13.29^*µ*3^
CALE 100 mg/kg	85.83 ± 8.55	199.50 ± 15.22^*β*3^	138.06 ± 16.56^*β*1 *µ*1^	84.33 ± 6.94^*µ*3^
CALE 200 mg/kg	86.17 ± 5.87	213.00 ± 7.51^*β*3^	137.00 ± 9.01^*β*2 *µ*3^	93.50 ± 5.98^*µ*3^
CALE 400 mg/kg	77.61 ± 7.23	211.72 ± 20.84^*β*3^	137.61 ± 14.28^*β*1 *µ*2^	81.94 ± 4.18^*µ*3^
GLC 5 mg/kg	81.44 ± 1.24	180.72 ± 8.75^*β*3^	82.83 ± 5.43^a1 *µ*3^	61.78 ± 8.59^a3 c1 *µ*3^

Each value represents mean ± SEM; n=6 for each treatment. ^**a**^Compared to the negative control, ^**c**^compared to CALE 200mg/kg, ^**β**^compared to baseline blood glucose level, and ^**µ**^compared to the blood glucose level at 30 minute. ^1^p < 0.05, ^2^p < 0.01, and ^3^p < 0.001. CALE = *Calpurnia aurea* leaf extract, DW = distilled water, and GLC = glibenclamide. Time refers to time after oral glucose loading.

**Table 3:** Antihyperglycemic activity of single dose of *Calpurnia aurea* leaf extract in STZ-induced diabetic mice.

**Group **	**Blood glucose level (mg/dl)**				
	**0 hr**	**2 hr**	**4 hr**	**6 hr**	**8 hr**
DW 10 ml/kg	394.11 ± 31.03	383.06 ± 27.65	396.39 ± 26.71	397.45 ± 18.52	399.61 ± 22.00
CALE 100 mg/kg	377.72 ± 43.57	364.39 ± 35.29	288.83 ± 51.29	261.33 ± 54.28	254.67 ± 48.16
CALE 200 mg/kg	314.39 ± 31.99	291.11 ± 38.52	254.78 ± 56.26	250.06 ± 51.84	246.50 ± 56.51
CALE 400 mg/kg	342.67 ± 58.07	313.39 ± 73.93	266.39 ± 53.99	290.00 ± 57.78	264.72 ± 56.88
GLC 5 mg/kg	368.50 ± 43.02	283.39 ± 39.09	176.61 ± 14.01^a1 *β*3^	171.72 ± 18.27^a1 *β*3^	155.72 ± 13.59^a2 *β*3^

Each value represents mean ± SEM; n=6 for each treatment. ^**a**^Compared to the negative control and ^**β**^compared to baseline blood glucose level. ^1^p < 0.05, ^2^p < 0.01, and ^3^p < 0.001. CALE = *Calpurnia aurea* leaf extract, DW = distilled water, and GLC = glibenclamide.

**Table 4:** Antihyperglycemic activity of repeated daily doses of *Calpurnia aurea* leaf extract in STZ-induced diabetic mice.

**Group **	**Fasting blood glucose level (mg/dl)**			**Percent reduction in baseline BGL**	
	**Baseline**	**7th day**	**14th day**	**7th day**	**14th day**
Diabetic control	394.11 ± 31.0^n3^	383.67 ± 45.83^n3^	387.00 ± 47.77^n3^	2.60%	1.80%
CALE 100 mg/kg	377.72 ± 43.57^n3^	251.39 ± 28.86^a1 n2^	250.44 ± 28.84^a1 *β*1 n1^	33.45%	33.69%
CALE 200 mg/kg	314.39 ± 31.99^n2^	243.56 ± 24.58^a1 n1^	239.94 ± 35.81^a1 n1^	22.53%	23.68%
CALE 400 mg/kg	342.67 ± 58.07^n3^	252.72 ± 14.43^a1 n2^	250.61 ± 14.42^a1 n1^	26.25%	26.87%
GLC 5 mg/kg	368.50 ± 43.02^n3^	145.56 ± 26.72^a3 *β*3^	136.67 ± 26.41^a3 *β*3^	60.49%	62.91%
Normal control	76.83 ± 2.51	77.67 ± 2.50	78.67 ± 2.75	-1.09%	-2.40%

Each value represents mean ± SEM; n=6 for each group. ^**a**^Compared to the diabetic control, ^**n**^compared to the normal control, and ^**β**^compared to baseline blood glucose level. ^1^p < 0.05, ^2^p < 0.01, and ^3^p < 0.001. CALE = *Calpurnia aurea* leaf extract and GLC = glibenclamide.

**Table 5:** Effect of repeated daily doses of the hydromethanolic leaf extract of *Calpurnia aurea* on body weight of STZ-induced diabetic mice.

**Group **	**Body weight (g)**			
	**Before induction of Diabetes**	**Baseline **	**7th day of treatment**	**14th day of treatment**
Diabetic control	28.67 ± 0.95	26.77 ± 0.89	23.88 ± 1.24^n2^	20.88 ± 1.15^n3 *β*2^
CALE 100 mg/kg	28.83 ± 1.09	27.98 ± 0.95	25.07 ± 1.06^n1^	23.78 ± 0.86^n3 *β*1^
CALE 200 mg/kg	28.83 ± 0.91	26.82 ± 1.18	24.83 ± 0.98^n1^	22.97 ± 0.76^n3 *β*1^
CALE 400 mg/kg	28.75 ± 0.96	27.70 ± 0.99	25.00 ± 1.02^n1^	23.25 ± 1.16^n3 *β*1^
GLC 5 mg/kg	28.75 ± 0.48	26.07 ± 0.73	25.95 ± 0.71	26.53 ± 0.72^a2^
Normal control	29.00 ± 0.47	29.45 ± 0.36	30.03 ± 0.53	30.70 ± 0.59

Each value represents mean ± SEM; n=6 for each group. ^**a**^Compared to the diabetic control, ^**n**^compared to the normal control, and ^**β**^compared to baseline body weight. ^1^p < 0.05, ^2^p < 0.01, and ^3^p < 0.001. CALE = *Calpurnia aurea* leaf extract and GLC = glibenclamide.

**Table 6:** Effect of repeated daily doses of hydromethanolic *Calpurnia aurea* leaf extract on serum lipid level of diabetic mice.

**Groups**	**Serum lipid level (mg/dl)**		
	**TC**	**TG**	**HDL-C**
Diabetic control	191.33±4.07^n3^	164.83±13.49^n3^	22.17±3.05^n3^
CALE 100 mg/kg	168.83±3.67^n3^	154.00±6.71^n3^	28.17±2.10
CALE 200 mg/kg	171.67±15.40^n3^	154.67±6.28^n3^	29.83±3.91
CALE 400 mg/kg	168.87±11.17^n3^	148.33±4.68^n3^	30.50±1.34
GLC 5 mg/kg	99.50±8.27^a3 b3 c3 d3^	77.50±5.55^a3 b3 c3 d3^	38.50±2.68^a2^
Normal control	83.83±5.36	73.50±7.26	41.17±4.88

Each value represents mean ± SEM; n=6 for each group. ^**a**^Compared to the diabetic control, ^**b**^compared to CALE 100mg/kg, ^**c**^compared to CALE 200 mg/kg, ^**d**^compared to CALE 400mg/kg, and ^**n**^compared to the normal control. ^1^p < 0.05, ^2^p < 0.01, and ^3^p < 0.001. CALE = *Calpurnia aurea* leaf extract, GLC = glibenclamide, TC = total cholesterol, TG = triglyceride, and HDL-C = high density lipoprotein cholesterol.

## Data Availability

All the data used to support the findings of this study are available from the corresponding author upon reasonable request.

## Abbreviations

ANOVA: Analysis of variance
BGL: Blood glucose level
CALE: * Calpurnia aurea* leaf extract
DM: Diabetes mellitus
DW: Distilled water
GLC: Glibenclamide
HDL: High density lipoprotein
HDL-C: High density lipoprotein cholesterol
IP: Intraperitoneal
LD50: Median lethal dose
LDL: Low density lipoprotein
OECD: Organization for Economic Cooperation and Development
STZ: Streptozotocin
TC: Total cholesterol
TG: Triglyceride
USD: United States Dollar.
